# Characteristics of subclinical seizures in an adult epilepsy population—a retrospective observational study

**DOI:** 10.3389/fneur.2026.1883867

**Published:** 2026-08-05

**Authors:** Lauren M. Lankhuijzen, Monique Cloherty, Joel S. Winston, Mark P. Richardson, Pedro F. Viana

**Affiliations:** 1 Department of Basic and Clinical Neuroscience, Institute of Psychiatry, Psychology and Neuroscience, King’s College London, London, United Kingdom; 2 Epilepsy Centre, Clinical Neurosciences Department, King’s College Hospital, London, United Kingdom

**Keywords:** epilepsy, neuropsychological assessment, presurgical, subclinical seizures, video-EEG

## Abstract

**Purpose:**

Subclinical seizures (SCSs) are commonly detected during video-EEG monitoring but frequently overlooked in clinical practice. Their prevalence, localization characteristics, and relationship to clinical seizures (CSs) and interictal epileptiform discharges (IEDs) remain incompletely defined. This study aimed to characterize SCS prevalence, localization, frequency, and concordance with CS and IED localization in a presurgical epilepsy cohort, and to examine associations between SCS presence and neuropsychological performance.

**Methods:**

A retrospective observational study was conducted in 116 adult patients undergoing inpatient scalp video-EEG telemetry and neuropsychological evaluation at King’s College Hospital between 2015 and 2023. SCS localization was compared with CS and IED localization using a five-category concordance framework and Cohen’s kappa for lateralization and temporal vs. extratemporal agreement.

**Results:**

SCSs were present in 26.7% of patients. No clinical or demographic variable was significantly associated with their presence. SCSs most frequently localized to left temporal and right extratemporal regions, whereas CSs were more evenly distributed across temporal foci. Full concordance between CS and SCS localization was observed in 55.2% of co-recorded studies; where incomplete, SCS localizations were more often contained within CS localizations than the reverse. IED localizations were more widespread than both seizure types. Lateralization agreement was moderate across all comparisons (*κ* = 0.40–0.47). SCS presence was not significantly associated with neuropsychological test scores.

**Conclusion:**

SCSs are present in approximately one quarter of presurgical epilepsy patients and show moderate concordance with CS and IED localization. Their distinct localization profile and frequent containment within CS zones support their value as complementary information in epilepsy care.

## 1 Introduction

Despite many decades of research and development, still up to a third of people with epilepsy (PWE) are resistant to available treatments and continue to suffer from recurrent seizures (1). Clinical approaches to the diagnosis and management of epilepsy are largely focused on detection of interictal activity and on detection and characterization of electroclinical seizures. Growing evidence points to an additional clinical relevance in subclinical seizure (SCS) monitoring (2–7).

SCSs are electrographic seizures with no subjective realization or overt, clinically observed somatic or neurological signs (8–10). They are commonly overlooked, as their detection requires prolonged video-EEG monitoring (4, 6), and their true prevalence and clinical significance is still somewhat debated (2, 5).

The characteristics of SCSs and their localization agreement with other findings on EEG, remain incompletely defined. While SCSs may reflect the same epileptogenic networks that generate clinical seizures (CSs), other evidence suggests they could also represent a physiologically distinct phenomenon (11, 12). These perspectives are not mutually exclusive. Uncertainty here has limited their integration into routine clinical decision-making, resulting in variability across clinicians in the management of these seizures (13).

Some evidence suggests that SCSs may predict epilepsy surgery outcomes, however, the literature is limited and inconsistent. One study (7), including 123 patients, found colocalization of SCS and CSs to be a favorable prognostic factor for post-operative seizure freedom. This has been mirrored in other smaller studies (11, 14), further finding that incomplete resection of the SCS onset zone is associated with significantly worse surgery outcomes. Meanwhile (2) found no evidence, in a cohort of 169 patients, that concordance between SCS and CS onset zone could predict surgical outcome, just that complete resection is most beneficial for good surgical outcome. Methodological differences and uncertainty around the physiological basis of SCSs continue to limit the understanding of its clinical significance in patient outcomes.

Real-time experimental video-EEG studies show that SCSs and also interictal epileptiform discharges (IEDs) can be associated with transient cognitive impairment (TCI) (30, 31). However, the longer-term implications of frequent SCSs on cognitive performance in the adult epilepsy population remains poorly understood. We identified this as an important area for consideration in understanding the clinical significance of SCSs.

Understanding the clinical significance of SCSs in PWE exceeds theoretical interests, as clinical decisions in epilepsy management can fall dependent on this question. Antiseizure medication have been found to significantly decrease SCS burden in PWE (15) and a subset of epilepsy patients with treated CSs continue to experience SCS activity, raising the debate of “treating the EEG, or the patient” (16).

The detection of SCSs is influenced by the recording modality used. Intracranial EEG techniques provide greater sensitivity for identifying focal epileptiform activity (2, 4). In contrast, scalp EEG has a more limited spatial resolution and reduced sensitivity to small or deep cortical activity (17). Here, reflecting current routine clinical practice, all recordings were obtained using scalp video-EEG; this approach ensured relevance to current routine clinical practices, while acknowledging its limitations in seizure detection sensitivity.

In this study, we aimed to describe (1) the prevalence of SCSs, (2) SCS localization/lateralization (3) SCS frequency and timing in relation to clinical seizures (4) SCS localization agreement with other findings on EEG, including clinical seizures and interictal epileptiform discharges (IEDs). We also performed a secondary analysis investigating whether variability in neuropsychology scores among adult epilepsy patients is significantly associated with SCS presence.

## 2 Materials and methods

### 2.1 Study type

The project operated under London South East Research Ethics Committee approval (reference 18/LO/2048) granted to the King’s Electronic Records Research Interface (KERRI).

### 2.2 Population

A retrospective observational study was conducted. We considered, for inclusion, all adult patients having underwent inpatient scalp video-EEG telemetry and neuropsychological evaluation at King’s College Hospital (KCH), for presurgical evaluation of epilepsy, between 2015 and 2023. The primary criteria for inclusion were as follows: (1) recording of at least one (clinical or subclinical) seizure or epileptiform discharges on video-EEG; (2) age between 18 and 70 years; (3) the completion of video-EEG telemetry and neuropsychological assessment within a 12-month period of one another; (4) a record of sufficient hearing, vision, concentration, and effort noted at the time of neuropsychological evaluation. We excluded patients with diagnosis of an epileptic encephalopathy (e.g., Lennox–Gastaut Syndrome), patients with severe intellectual disability characterised by full-scale intelligence quotient below 40 (18) and commensurate adaptive function, as well as patients who registered in the NHS Data Opt-Out (NDOO) scheme.

### 2.3 Demographic and clinical variables

Demographic data comprising the patient’s age in years and biological sex (male or female) were retrieved. Clinical variables, such as days of video EEG monitoring in telemetry, previous resective epilepsy surgery, age of onset (years) epilepsy duration (years), number of antiseizure medications (ASMs) and handedness (right and non-right-handed) were noted. Magnetic Resonance Imaging (MRI) findings were classified as: malformation of cortical development (including focal cortical dysplasia and others), post-traumatic, post-stroke, post-infectious, tumors, hippocampal sclerosis, vascular malformation, non-specific encephalomalacia, encephalocele, inflammatory and non-lesional.

### 2.4 Data from video-EEG telemetry recordings

We collected information about the presence and localization of clinical seizures, subclinical seizures and interictal epileptiform discharges from original clinician reports. SCSs were defined as ictal activity with clear spatiotemporal evolution, unreported by the patient and with no observed clinical signs, including subtle changes such as behavior arrest or arousal from sleep.

The localization of CS, SCS and IEDs was classified at the level of individual foci, each assigned to one of the following categories: left temporal, right temporal, left extratemporal, right extratemporal, left diffuse, right diffuse, or bilateral/diffuse. Recordings could involve more than one focus independently. Localization was then characterised at two levels. At the recording level, each recording was assigned a localization pattern defined by the combination of all active foci (e.g., left temporal only, or right temporal + left extratemporal), and the frequency of each pattern was summarized descriptively. At the focus level, the proportion of recordings involving each individual focus was reported independently, such that recordings with multiple foci contributed to more than one category.

Clinical EEG data were acquired at King’s College Hospital Department of Neurophysiology in unshielded and artificially lit rooms. Twenty-one silver disk scalp electrodes were placed according to the Modified Maudsley Configuration. This is similar to the 10–20 system, with slightly different positioning (∼20 mm lower) of the temporal electrodes chain for optimal frontal and temporal coverage (19). The reference channel was typically placed on the midline anterior to Pz. Single-channel ECG was simultaneously recorded. EEG data were acquired using the Xltek system (Natus, 32-channel, sampling rate 500 Hz).

### 2.5 Neuropsychological evaluation

At KCH, cognitive assessment of PWE is systematically performed with the Wechsler Abbreviated Scale of Intelligence, 2nd Edition (WASI-II) and Wechsler Memory Scale, 4th edition (WMS-IV). These batteries can frequently detect problems with higher-order, top-down mental processes needed for attention, response inhibition, memory and concept formation (20, 21). However, an overall score such as that expressed by the Full-Scale Intellectual Quotient (FSIQ), appear to be less effective in PWE, as a reduced processing speed or working memory impairment can obscure interpretations of the cognitive impairment (CI) (22). The WASI-II also has additional easier and harder items to better capture performance at the very low and very high ends of ability. Therefore, the WASI-II scores for the Verbal Comprehension Index (VCI) and Perceptual Reasoning Index (PRI) and the WMS-IV score for the Auditory Memory Index (AMI) were deemed the most appropriate measurements for primary analysis – as these are well-established, transferable, reliable, and suited to detecting common CIs in PWE (21).

Further intelligence scores derived for exploratory analysis included the Full-Scale Intellectual Quotient (FSIQ) and scaled age-normed scores for block design, vocabulary, matrix reasoning, and similarities subtests. The Hospital Anxiety and Depression Scale (HADS) was used to measure the symptoms of anxiety and depression. When this was unavailable from records (*n* = 7), results from the Patient Health Questionnaire and Generalized Anxiety Disorder 7 were extracted instead. Neuropsychological evaluation was not conducted during seizures.

### 2.6 Statistical analysis

The following tests were used in analysis of demographic and basic clinical characteristics: Pearson’s Chi-squared test (for comparing two categorical variables), independent samples *t*-test or Mann–Whitney-*U* test (for comparing a continuous variable between two groups).

IED, CS and SCS localization patterns were visualized using UpSet plots, which display the frequency of all observed multi-focus combinations alongside the marginal frequency of each individual focus. This approach was preferred over Venn diagrams given the number of possible focus combinations.

Concordance between the localization patterns of CS, SCS and IEDs was assessed for all three pairwise comparisons (CS vs. SCS, CS vs. IED, SCS vs. IED). For each pair, recordings in which both categories were simultaneously present were classified into one of five concordance categories: full concordance (identical localization patterns), first pattern contained within the second, second pattern contained within the first, partial overlap (shared and non-shared foci), or full discordance (no shared foci).

The same five-category concordance framework, the categorical lateralization (left, right, bilateral, diffuse) and localization-type (temporal, extratemporal, mixed/diffuse) agreement analyses quantified with Cohen’s *κ*, were additionally applied to compare CS, SCS and IED localization with the localization of MRI-identified putative epileptogenic lesions. As these comparisons require a localizable structural lesion, they were restricted to patients with a lesional MRI and are reported in Supplementary material.

To quantify agreement at a categorical level, localization patterns were collapsed into two simplified classification schemes: lateralization (left, right, bilateral, or diffuse) and localization type (temporal, extratemporal, or mixed/diffuse). Agreement between CS, SCS and IEDs for each classification scheme was quantified using Cohen’s kappa (*κ*) and was visualized as confusion matrices. Statistical analysis was performed using R Studio (2025.09.2 + 418), with a significance level of *p* < 0.05.

## 3 Results

### 3.1 Clinical and demographic characteristics

**Figure 1:**
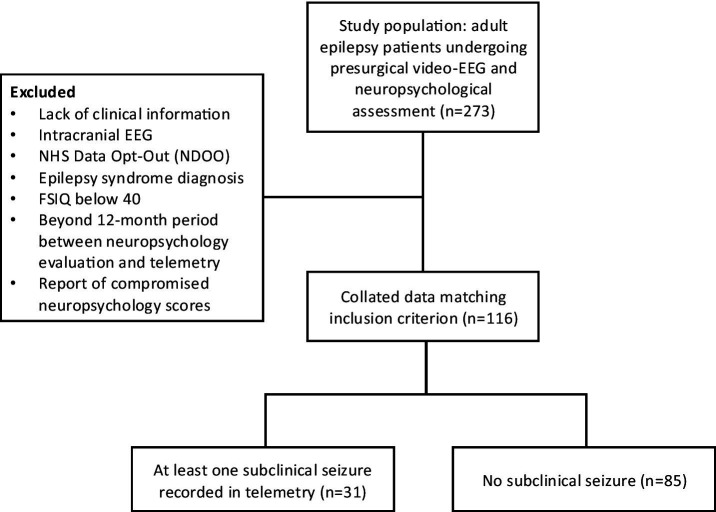
Patient selection and inclusion overview.

Out of a total of 273 patients, 116 met the inclusion criteria for analysis (Figure 1). SCSs were recorded in 26.7% of included patients (standard error = 4.1, 95% confidence interval = 18.7 to 34.8%). Patient age ranged from 18 to 70, with a mean age of 40 (SD 13.6) years. Most patients (86.2%) did not have a history of previous resective epilepsy surgery. The most reported MRI lesions were hippocampal sclerosis and malformations of cortical development (both at 18.1%). None of the main demographic and clinical characteristics were significantly associated with the presence of subclinical seizures (Table 1).

**Table 1:** Demographic and basic clinical characteristics of the cohort, comparing groups with and without subclinical seizures.

Variable	Total cohort (*n* = 116)	No subclinical seizures (SCS−; *n* = 85)	Subclinical seizures (SCS+; *n* = 31)	Significance (SCS+ vs. SCS−)
Age, mean (SD)	39.78 (13.60)	40.01 (13.36)	39.13 (14.44)	*p* = 0.768‡
Days in telemetry, median (IQR)	5.50 (4.00–7.00)	5.00 (4.00–7.00)	6.00 (5.00–7.50)	*p* = 0.082#
Sex, *n* (%)				
Male	64 (55.2%)	51 (60.0%)	13 (41.9%)	*p* = 0.176†
Female	52 (44.8%)	34 (40.0%)	18 (58.1%)	
Previous resective epilepsy surgery, *n* (%)				
No	100 (86.2%)	76 (89.4%)	24 (77.4%)	*p* = 0.287†
Yes	16 (13.8%)	9 (10.6%)	7 (22.6%)	
Handedness, *n* (%)				
Right-handed	96 (82.8%)	72 (84.7%)	24 (77.4%)	*p* = 0.358†
Non-right-handed	20 (17.2%)	13 (15.3%)	7 (22.6%)	
Age of onset (years)	16.24 (12.57)	15.82 (11.44)	17.35 (15.32)	*p* = 0.614‡
Epilepsy duration (years)	23.58 (15.21)	24.26 (15.31)	21.77 (15.04)	*p* = 0.439‡
Number of ASMs (current), median (IQR)	2.00 (2.00–3.00)	2.00 (1.00–3.00)	3.00 (2.00–3.00)	*p* = 0.614#
Clinical seizure frequency, *n* (%)				
Less than daily	71 (61.2%)	52 (61.2%)	19 (61.3%%)	*p* = 1.00†
At least daily	45 (38.8%)	33 (38.8%%)	12 (38.7%)	
MRI lesion present, *n* (%)	71 (61.2%)	55 (64.7%)	16 (51.6%)	*p* = 0.088†
MRI lesion type, *n* (%)				
Hippocampal sclerosis	21 (18.1%)	17 (20.0%)	4 (12.9%)	
Malformation of cortical development	21 (18.1%)	15 (17.6%)	6 (19.4%)	
Tumor	12 (10.3%)	10 (11.8%)	2 (6.5%)	
Vascular malformation	4 (3.4%)	2 (2.4%)	2 (6.5%)	
Post-infectious	4 (3.4%)	3 (3.5%)	1 (3.2%)	
Post-traumatic	4 (3.4%)	3 (3.5%)	1 (3.2%)	
Encephalomalacia	2 (1.7%)	1 (1.2%)	1 (3.2%)	
Encephalocele	1 (0.9%)	1 (1.2%)	0 (0.0%)	
Inflammatory	1 (0.9%)	0 (0.0%)	1 (3.2%)	
Post-stroke	1 (0.9%)	1 (1.2%)	0 (0.0%)	

Significant difference at *p* < 0.05; †Pearson’s Chi-squared test; ‡Independent samples *t*-test; #Mann–Whitney-U non-parametric test. Statistical analysis could not be performed on MRI lesion type because groups were too small. SD – standard deviation; n – number. Variable ‘days in telemetry’ refers to the number of consecutive days of EEG monitoring in telemetry.

Among the 26.7% of patients with SCSs present in telemetry recordings (Table 2), 45.2% experienced them at least once daily. SCSs occurred in the recording prior to the appearance of clinical seizures in 61.3% of patients and occurred after ASM reduction in 61.3% of patients.

**Table 2:** Frequency and timing of subclinical seizures in the SCS + group.

SCS Variable	n (%)
Subclinical seizure frequency	
At least daily	14 (45.2%)
Less than daily	17 (54.8%)
Subclinical seizures occurred before clinical seizures	
Yes	19 (61.3%)
No	12 (38.7%)
Subclinical seizures occurred before antiseizure medication reduction	
Yes	12 (38.7%)
No	19 (61.3%)

Values are presented as number of total patients (percentage) within the SCS+ group (*n* = 31). SCS = subclinical seizures. AED = antiepileptic drug.

### 3.2 Localization patterns

**Figure 2:**
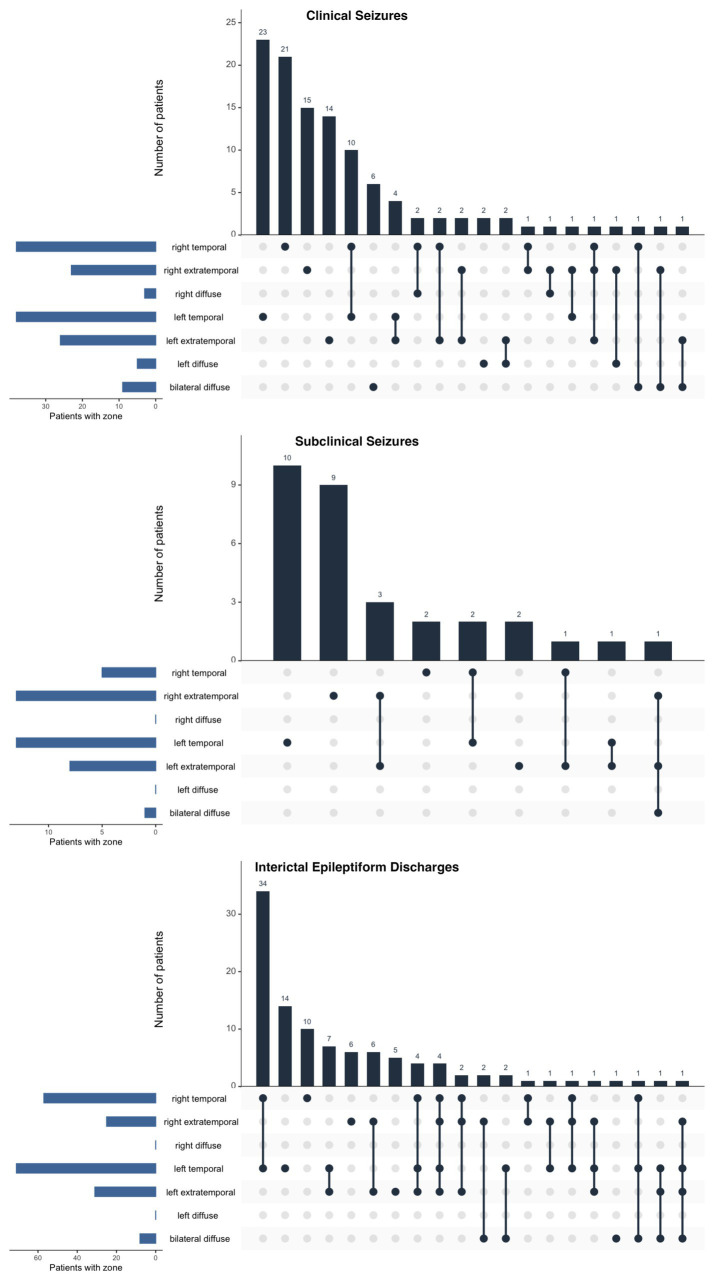
Localization patterns of clinical seizures, subclinical seizures, and interictal epileptiform discharges. UpSet plots displaying the frequency of localization patterns recorded during video-EEG monitoring for clinical seizures (top), subclinical seizures (middle), and interictal epileptiform discharges (bottom). Each column represents a distinct combination of simultaneously active foci, with filled circles indicating which foci are included and connecting lines denoting co-occurring foci. Vertical bars indicate the number of recordings with each combination (intersection size). Horizontal bars on the left indicate the total number of recordings involving each individual focus, irrespective of co-occurring foci.

Regarding clinical seizure localization (Figure 2), the most frequent localization patterns were left temporal only (*n* = 23, 20.7%), right temporal only (*n* = 21, 18.9%), right extratemporal only in (*n* = 15, 13.5%) and left extratemporal only (*n* = 14, 12.6%). Bilateral temporal seizures were recorded in 10 studies (9.0%). At the focus level, left and right temporal involvement were equally frequent (each *n* = 38, 34.2%), followed by left extratemporal (*n* = 26, 23.4%) and right extratemporal (*n* = 23, 20.7%) foci.

In contrast, the most frequent localization patterns for subclinical seizures were left temporal only (n = 10, 32.3%) and right extratemporal only (n = 9, 29.0%), together accounting for 61.3% of recordings. At the focus level, right extratemporal and left temporal foci were the most commonly involved regions (each n = 13, 41.9%), followed by left extratemporal (n = 8, 25.8%) and right temporal (n = 5, 16.1%) foci. Compared with clinical seizures, right extratemporal foci were more frequent and right temporal foci less frequent.

Regarding IEDs, the most frequent localization pattern was bilateral temporal (right temporal + left temporal), observed in 34 studies (32.7%), followed by left temporal only (*n* = 14, 13.5%) and right temporal only (n = 10, 9.6%). At the focus level, left temporal foci were the most frequent (*n* = 71, 68.3%), followed by right temporal (n = 57, 54.8%), left extratemporal (*n* = 31, 29.8%), and right extratemporal (*n* = 25, 24.0%) foci.

### 3.3 Concordance between patterns

Concordance between localization patterns was assessed in studies where two categories were simultaneously recorded: CS with SCS (*n* = 29), CS with IED (*n* = 101), and SCS with IED (*n* = 29)–Table 3.

**Table 3:** Concordance between clinical seizures, subclinical seizures and IEDs.

Concordance	CS vs IED (*n* = 101)	CS vs SCS (*n* = 29)	SCS vs IED (*n* = 29)
Full concordance	39 (38.6%)	16 (55.2%)	12 (41.4%)
First zone contained within second zone	39 (38.6%)	2 (6.9%)	11 (37.9%)
Second zone contained within first zone	3 (3%)	4 (13.8%)	1 (3.4%)
Partial overlap	8 (7.9%)	3 (10.3%)	1 (3.4%)
Fully discordant	12 (11.9%)	4 (13.8%)	4 (13.8%)

Localization patterns were compared using a five-category framework. Values are presented as number of patients with percentages in parentheses.

Full concordance between clinical and subclinical seizures was observed in 16 of 29 studies (55.2%). Where concordance was incomplete, SCS localizations were more often contained within the CS localization than the reverse (4 vs. 2 studies). Partial overlap and full discordance were each present in a minority of studies (10.3 and 13.8% respectively).

Full concordance between CS and IED was observed in 39 of 101 studies (38.6%). In non-concordant studies, CS localizations were more frequently contained within the IED localization than the reverse (39 vs. 3 studies).

Full concordance between SCS and IED was observed in 12 of 29 studies (41.4%). Similarly, SCS localizations were more often contained within the IED localization than the reverse (11 vs. 1 study). Partial overlap and full discordance were each present in a minority of studies (3.4 and 13.8% respectively).

**Figure 3:**
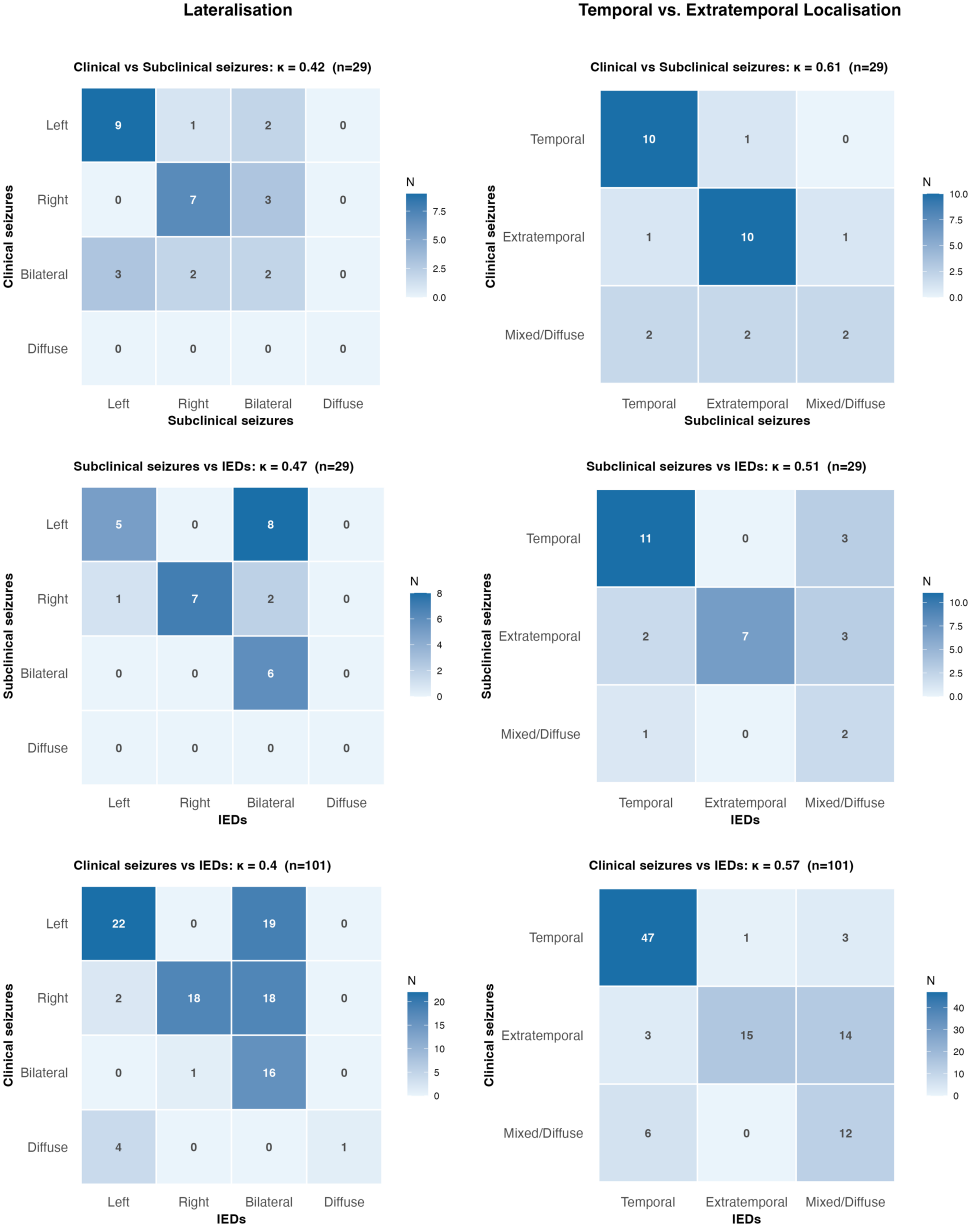
Agreement in lateralization and temporal vs. extratemporal localization between clinical seizures, subclinical seizures, and interictal epileptiform discharges. Confusion matrices displaying categorical agreement in lateralization (left column) and temporal vs. extratemporal localization (right column) between pairwise combinations of clinical seizures vs subclinical seizures (top row), subclinical seizures vs interictal epileptiform discharges (middle row), and clinical seizures vs interictal epileptiform discharges (bottom row). Localization patterns were collapsed into lateralization categories (left, right, bilateral, diffuse) and localization type categories (temporal, extratemporal, mixed/diffuse). Cell values indicate the number of recordings. Cohen's kappa (κ) and the number of recordings included in each comparison (n) are shown above each matrix. Only recordings in which both categories were simultaneously captured are included.

Concordance between CS, SCS and IEDs with regards to lateralization and temporal vs. extratemporal localization was assessed at the recording level and is illustrated in Figure 3. Lateralization agreement was moderate across all three comparisons (CS vs. SCS: *κ* = 0.42; CS vs. IED: κ = 0.40; SCS vs. IED: *κ* = 0.47). Unilateral concordance was generally preserved, while bilateral IEDs showed frequent association with both unilateral and bilateral seizure onsets. Agreement for temporal vs. extratemporal localization was similar (CS vs. SCS: *κ* = 0.61; CS vs. IED: κ = 0.57; SCS vs. IED: κ = 0.51), with the strongest agreement observed for the CS vs. IED comparison.

Concordance between electrophysiological and structural (MRI lesion) localization was additionally assessed in patients with a localizable lesion (CS vs. MRI, *n* = 67; SCS vs. MRI, *n* = 16; IED vs. MRI, *n* = 63; Supplementary Figures 1, 2 and Supplementary Table 1). Full concordance with the MRI lesion was observed in 38.8% (26/67) of clinical seizures, 31.2% (5/16) of subclinical seizures and 30.2% (19/63) of IEDs, with at least some spatial overlap (full concordance, containment, or partial overlap) in 59.7, 50.0 and 66.7% of comparisons, respectively. In keeping with the wider distribution of IEDs noted above, the MRI lesion was frequently contained within a broader interictal field (33.3% of cases).

### 3.4 Association between SCS and neuropsychological scores

A secondary analysis considering whether variability in neuropsychology scores among adult epilepsy patients was significantly associated with SCS presence found no significant associations (Table 4).

**Table 4:** Neuropsychological scores in adult epilepsy patients with and without subclinical seizures.

Neuropsychology scores	No subclinical seizures (*n* = 90) median (IQR)	Subclinical seizures present (*n* = 31) median (IQR)	*p*-value*
HADS–Hospital anxiety and depression scale	8.63 (5.07)	7.35 (3.95)	*p*=0.227*‡*
VCI–Verbal comprehension index	91.63 (14.81)	88.10 (11.20)	*p*=0.182*‡*
PRI–Perceptual reasoning index	92.24 (16.22)	90.61 (18.07)	*p*=0.673*‡*
FSIQ–Full scale intelligence quotient	88.59 (13.79)	90.90 (13.61)	*p*=0.509*‡*
AMI–Auditory memory index	85.99 (18.98)	88.08 (17.99)	*p*=0.631*‡*
Block design	8.46 (3.19)	8.13 (3.17)	*p*=0.625*‡*
Matrix reasoning	8.84 (3.35)	9.32 (3.38)	*p*=0.505*‡*
Vocabulary	8.85 (2.99)	8.31 (2.61)	*p*=0.358*‡*
Similarities	8.21 (2.98)	7.83 (2.27)	*p*=0.482*‡*

Significant difference at *p* < 0.05; ‡Independent samples *t*-test; scores are presented as the median number with interquartile range (IQR).

## 4 Discussion

### 4.1 Prevalence of subclinical seizures

Subclinical seizures were present in 26.7% of patients in our epilepsy cohort. This is higher than some other retrospective video EEG studies, including a similar size focal-epilepsy cohort reporting 9.76% of SCSs (4), a larger drug resistant cohort reporting 2.9% (3), and a broader epilepsy-center population reporting 5.3% (23). Our patients underwent an average of 5.92 days of monitoring (longer than studies with lower prevalence reports), and longer recording duration has been associated with increased SCS detection (6). In addition, our cohort consisted exclusively of patients undergoing presurgical evaluation, representing a more drug-resistant population than that of (23) and following a different criterion to (4), which may partly explain the higher yield. The use of a single consecutive cohort may also reduce sampling variability. Consequently, our prevalence rate should be interpreted as characteristic of a severe, pre-surgical scalp video-EEG population rather than the broader epilepsy community.

All recordings were obtained using scalp video-EEG, which is less sensitive in SCS detection than intracranial EEG monitoring. Intracranial studies report SCSs in up to 50% of patients (2, 4) and only about 10% of intracranial cortical spikes with a source area under 10 cm^2^ are detectable on the scalp (17).

Neither the aetiology of epilepsy nor any other clinical or demographic variable examined was significantly associated with the presence of SCSs, consistent with previous prevalence studies (2, 5). These null findings should be interpreted with caution given the sample size, though the cohort was broadly representative of comparable presurgical epilepsy populations (3, 4, 23).

### 4.2 Subclinical seizure localization and lateralization

The localization of SCSs in our cohort differed from that of CSs, with SCSs more frequently involving the left temporal and right extratemporal regions, whereas CSs were more evenly distributed between left and right temporal foci. The higher frequency of right extratemporal SCSs compared to CSs may be explained by the tendency of certain extratemporal regions — particularly parietal and posterior cortices — to generate ictal activity that remains clinically silent until propagating to symptomatogenic regions such as the temporal lobe. The ictal onset zone in parietal lobe epilepsy can be clinically and electrographically silent at scalp EEG, with seizures becoming manifest only after propagating to another brain region such as the temporal, occipital, or frontal lobes (24). This may result in extratemporal seizures being more likely to be captured as subclinical rather than clinical events on scalp video-EEG. Where concordance was incomplete, SCS localizations were more often contained within CS localizations than the reverse, suggesting that CSs tend to involve a broader or more propagated network than SCSs at the time of clinical expression. The overall moderate concordance between SCS and CS localization (full concordance 55.2%) is broadly consistent with prior scalp video-EEG studies (2, 7), supporting the view that SCS localization provides complementary information to CS localization in presurgical evaluation.

### 4.3 Subclinical seizure frequency and timing in relation to clinical seizures

Nearly half of patients with SCSs in our cohort experienced them on a daily basis during telemetry (≈45.2%), more often than CS, aligning with other observations that subclinical activity occurs more often than clinical seizures during monitoring (2, 14).

Subclinical seizures occurred prior to the recording of clinical seizures in most patients (61.3%), consistent with earlier findings suggesting that subclinical discharges may sometimes represent early or partial activation within the same epileptogenic network (2, 8). This arises the question whether SCSs may act as electrophysiological precursors of clinical seizures, marking periods of heightened cortical excitability.

The observation that SCSs occurred more frequently after AED reduction (61.3%) supports their pharmacological sensitivity, corroborating reports that medication tapering may increase both clinical and subclinical seizure occurrence (25). This suggests that SCSs may respond dynamically to therapeutic modulation and could serve as indicators of cortical excitability under reduced drug levels.

### 4.4 Subclinical seizure localization agreement with other findings on EEG, including clinical seizures and interictal discharges

The moderate agreement we observed between SCSs, IEDs and CSs is consistent with prior work showing that SCSs frequently originate from the same cortical region as clinical seizures (4). However, the remainder of SCSs which were not considered to be concordant with IEDs, or CSs are not insignificant.

Our cohort demonstrated stronger agreement for lobar (temporal vs. extratemporal) localization of SCSs, IEDs and CSs than for hemispheric lateralization. A similar pattern, higher lobar than lateralizing accuracy, has been reported for clinical seizure semiology (26).

Concordance of CS, SCS and IED localization with the structural MRI lesion broadly paralleled the electrophysiological comparisons, including the tendency for interictal discharges to extend beyond, and contain, the lesion; however, the small number of lesional cases, particularly for subclinical seizures (*n* = 16), limits interpretation, and these analyses are reported only as Supplementary material.

### 4.5 Secondary analysis: the effect of subclinical seizure presence on neuropsychological test scores

In this study, the presence of SCSs was not significantly associated with any neuropsychological test scores. While this relationship has not previously been examined in an adult epilepsy cohort, our findings contrast with some pediatric studies suggesting long-term cognitive impact of SCSs (27, 28). It is possible that in adults, the presence of SCSs is less informative than their characteristics, such as localization, frequency, or propagation patterns, which may better reflect the complexity of underlying epileptogenic networks. This interpretation aligns with evidence from Tsuboyama et al. (5), who found no association between SCS presence and postoperative cognitive outcomes in a pediatric cohort undergoing intracranial EEG monitoring (5). However, the current findings should be interpreted with caution. The sample size was modest, and the cohort was heterogeneous in terms of epilepsy type, medication load, and duration.

### 4.6 Study limitations

The retrospective design of this study introduced some limitations in the quantification of SCS frequency, as clinical reports were at times qualitative rather than numerical, and access to the full dataset was not always available for prolonged recordings. This resulted in an underestimation of SCS frequency in some patients. The definition of SCSs may also be a matter of debate. The distinction between CSs and SCSs relies on clinical observation, and it is possible that subtle behavioral changes associated with ictal activity were not detected, particularly in the absence of formal neurological testing at the time of each event.

The neuropsychological findings should be interpreted in the context of several limitations. The cohort, whilst broadly representative of presurgical epilepsy populations, was heterogeneous in terms of aetiology, seizure type, and localization, which may have reduced statistical power to detect group differences. In addition, the lack of association between SCS presence and neuropsychological outcomes could reflect limitations of scalp EEG, which, may not detect SCS arising from deeper areas of the cortex. Medication reduction during video-EEG monitoring is standard practice but is a known confounder of cognitive performance, and its potential effects on neuropsychological results cannot be excluded. Due to this being a single center study, the sample size was also limited, future work would benefit from a multi-center analysis to increase the sample size.

A further limitation of this study is that postoperative outcomes were not assessed. Our cohort was not specifically selected based on surgical outcome, and available follow-up data were limited due to variability in timing of surgery, postoperative imaging, and some patients undergoing evaluation without subsequent surgery at our center. Therefore, prospective studies are needed to further examine the prognostic value of SCSs and their characteristics in epilepsy surgery.

### 4.7 Implications and further research

Future studies should aim to prospectively characterize the longitudinal occurrence patterns of subclinical seizures in patients with focal epilepsy. A key unresolved question is whether subclinical and clinical seizures represent manifestations of the same underlying epileptic process and covary over time, or whether they can evolve independently, for instance, persisting or emerging in the absence of clinical seizures following treatment changes. Understanding this relationship has implications for how seizure burden is defined and monitored clinically, as current practice largely relies on patient-reported clinical events.

Whether subclinical seizure frequency constitutes a meaningful marker of epilepsy severity, distinct from or complementary to clinical seizure burden, also warrants prospective investigation. If subclinical seizures track epilepsy activity independently of clinical seizures, they may provide additional prognostic information, particularly in patients with well-controlled clinical seizures in whom ongoing subclinical ictal activity might otherwise go undetected. This raises the question of whether systematic monitoring of subclinical seizures over time, for example using ambulatory or long-term EEG (29), would be of clinical value in selected patient groups.

## Data Availability

The raw data supporting the conclusions of this article will be made available by the authors, without undue reservation.
