# Supplementary material for: Characteristics of subclinical seizures in an adult epilepsy population—a retrospective observational study
Source: Front Neurol. 2026 Aug 5;17:1883867. doi: 10.3389/fneur.2026.1883867 (PMC13487370; doi:10.3389/fneur.2026.1883867)
Supplement: Supplementary file 1 [file Supplementary_file_1.DOCX]

**Supplementary Material**

**Supplementary Figure 1.** Localisation of MRI-identified putative epileptogenic lesions. UpSet plot displaying the frequency of lesion localisation patterns; vertical bars indicate the number of patients with each combination of involved zones, and horizontal bars the number of patients involving each individual zone, irrespective of co-occurring zones.

**Supplementary Figure 2.** Agreement in lateralisation (left column) and temporal vs. extratemporal localisation (right column) between MRI lesion localisation and clinical seizures (top row), subclinical seizures (middle row) and interictal epileptiform discharges (bottom row). Localisation patterns were collapsed into lateralisation categories (left, right, bilateral, diffuse) and localisation-type categories (temporal, extratemporal, mixed/diffuse). Cell values indicate the number of patients; Cohen's κ and the number of patients included (n) are shown above each matrix. Only patients with both a localisable lesion and the relevant electrophysiological localisation are included.

| Category | CS vs MRI (N=67) | IED vs MRI (N=63) | SCS vs MRI (N=16) |
| --- | --- | --- | --- |
| Full concordance | 26 (38.8%) | 19 (30.2%) | 5 (31.2%) |
| First zone contained within the second | 5 (7.5%) | 0 (0%) | 1 (6.2%) |
| Second zone contained within the first | 7 (10.4%) | 21 (33.3%) | 2 (12.5%) |
| Partial overlap | 2 (3%) | 2 (3.2%) | 0 (%) |
| Discordant | 27 (40.3%) | 21 (33.3%) | 8 (50%) |

**Supplementary Table 1.** Concordance between clinical seizures, subclinical seizures and IEDs and MRI lesion localisation, using the same five-category framework as Table 3 (full concordance; first zone contained within second zone; second zone contained within first zone; partial overlap; fully discordant). Values are number of patients with percentages in parentheses; N denotes patients with both a localisable lesion and the relevant electrophysiological localisation.
